# Effect of Annealing Temperature on Spatial Atomic Layer Deposited Titanium Oxide and Its Application in Perovskite Solar Cells

**DOI:** 10.3390/nano10071322

**Published:** 2020-07-05

**Authors:** Chia-Hsun Hsu, Ka-Te Chen, Pao-Hsun Huang, Wan-Yu Wu, Xiao-Ying Zhang, Chen Wang, Lu-Sheng Liang, Peng Gao, Yu Qiu, Shui-Yang Lien, Zhan-Bo Su, Zi-Rong Chen, Wen-Zhang Zhu

**Affiliations:** 1 School of Opto-electronic and Communication Engineering, Xiamen University of Technology, Xiamen 361024, China; chhsu@xmut.edu.cn (C.-H.H.); kachen-123@163.com (K.-T.C.); xyzhang@xmut.edu.cn (X.-Y.Z.); chenwang@xmut.edu.cn (C.W.); asd2324884146@163.com (Z.-B.S.); s773951741@163.com (Z.-R.C.); wzzhu@xmut.edu.cn (W.-Z.Z.); 2 School of Information Engineering, Jimei University, Xiamen 361021, China; ph.huang@jmu.edu.cn; 3 Department of Materials Science and Engineering, Da-Yeh University, Changhua 51591, Taiwan; wywu@mail.dyu.edu.tw; 4 CAS Key Laboratory of Design a Assembly of Functional Nanostructures, and Fujian Provincial Key Laboratory of Nanomaterials, Fujian Institute of Research on the Structure of Matter, Chinese Academy of Sciences, Fuzhou 350002, China; lushengliang@fjirsm.ac.cn (L.-S.L.); peng.gao@fjirsm.ac.cn (P.G.); 5 Key Laboratory of Green Perovskites Application of Fujian Province Universities, Fujian Jiangxia University, Fuzhou 350108, China; yuqiu@fjjxu.edu.cn; 6 Fujian Key Laboratory of Optoelectronic Technology and Devices, Xiamen University of Technology, Xiamen 361024, China

**Keywords:** spatial atomic layer deposition, titanium dioxide, annealing, electron transport layer, perovskite

## Abstract

In this study, spatial atomic layer deposition (sALD) is employed to prepare titanium dioxide (TiO_2_) thin films by using titanium tetraisopropoxide and water as metal and water precursors, respectively. The post-annealing temperature is varied to investigate its effect on the properties of the TiO_2_ films. The experimental results show that the sALD TiO_2_ has a similar deposition rate per cycle to other ALD processes using oxygen plasma or ozone oxidant, implying that the growth is limited by titanium tetraisopropoxide steric hindrance. The structure of the as-deposited sALD TiO_2_ films is amorphous and changes to polycrystalline anatase at the annealing temperature of 450 °C. All the sALD TiO_2_ films have a low absorption coefficient at the level of 10^−3^ cm^−1^ at wavelengths greater than 500 nm. The annealing temperatures of 550 °C are expected to have a high compactness, evaluated by the refractive index and x-ray photoelectron spectrometer measurements. Finally, the 550 °C-annealed sALD TiO_2_ film with a thickness of ~8 nm is applied to perovskite solar cells as a compact electron transport layer. The significantly enhanced open-circuit voltage and conversion efficiency demonstrate the great potential of the sALD TiO_2_ compact layer in perovskite solar cell applications.

## 1. Introduction

Among a wide variety of metal oxides, TiO_2_ is a promising material for many emerging applications, such as gas sensors [1,2], dye-sensitized solar cells [3,4], photocatalysis [5] and gate insulators in metal-oxide-semiconductor field-effect transistors [6]. The characteristics of TiO_2_ thin films prepared by sol-gel, chemical vapor deposition or sputtering have been extensively studied [7,8,9,10]. Fujishima et al. reviewed the properties of TiO_2_ prepared by various methods, the fundamentals of photocatalysts, as well as applications [11]. Carp et al. reviewed the photoinduced reactivity of TiO_2_ [12]. In recent years, atomic layer deposition (ALD) has received great attention due to its unique features, such as the ability to prepare highly conformal thin films on high-aspect ratio substrates and to control the film thickness on an atomic level [13,14]. The self-limiting surface reaction in ALD leads to a continuous pinhole-free film, which can be used to deposit different metals, metal oxides and nitrides at relatively low temperatures [15,16,17]. Due to the high density of ALD thin films, single-layer and multi-layer stacks of TiO_2_ by thermal- and plasma-assisted ALD have been used for packaging and moisture-proof purposes [18,19]. The ALD TiO_2_ is also expected to be a promising electron transport layer (ETL) of perovskite solar cells owing to its high compactness and ability to have excellent coverage on a transparent conductive layer [20,21,22,23]. ALD TiO_2_ films can be deposited by using different titanium precursors, such as TiCl_4_ [24], TiI_4_ [25], tetra-dimethyl-amino titanium [26], titanium tetraisopropoxide (TTIP) [27], titanium ethoxide [28] and titanium methoxide [29]. Details for the preparation of TiO_2_ films based on these precursors are reviewed in ref. [30]. Among the various precursors, TTIP is cost-effective and has the highest vapor pressure, which makes it an interesting precursor for the ALD process development. For the metal precursors with a low vapor pressure, such as tetrakis(dimethylamido)titanium, the saturation of the surface chemical reaction can hardly be obtained unless by using a high bubbler temperature, high flow rate of the carrier gas and slow substrate moving speed. In comparison, by using a high vapor pressure precursor such as TTIP, a lower bubbler temperature and a faster substrate speed can be used.

Metal oxides often require an annealing process to improve the density of the film, and the crystal structure of the film is strongly affected by the annealing temperature. The existing research on TiO_2_ annealing is mostly based on sol-gel [8,31] or sputtering [32], where TiO_2_ is reported to have mainly three types of crystal phases, an anatase phase at below 600 °C, rutile phase at above 800 °C and brookite mesophase. The crystal phase of TiO_2_ also depends on the particle size, due to the interplay between the thermodynamic quantities, particularly the surface energy [33]. Although this is usually a concern for nanoparticles, nanocrystalline particles could be formed in the ALD films. Nabatame et al. [34] studied anatase TiO_2_ films fabricated by using ALD and postdeposition annealing, and discussed the flat band voltage change caused by the bottom interface dipole as well as how the dipole relates to oxygen introduced into the TiO_2_ layer during oxidation annealing. Few studies reported the effect of annealing on the properties of plasma or thermal ALD TiO_2_ films. Luka et al. studied the mechanism of the crystallization of thermal ALD TiO_2_ based on TiCl_4_/H_2_O annealed at 160–220 °C [35]. Matsui et al. reported that TiO_*x*_ can act as either an electron or hole selective contact depending on the thermal or plasma ALD process and that its selectivity is strongly affected by post-annealing and the work function of the metal or transparent conducting oxide contact on top of the TiO_*x*_ layer [36]. Won et al. investigated the effect of post-annealing in vacuum on the electrical properties and interfacial reaction in a thermal ALD TiO_2_/SiO_2_/Si system [37]. However, there are very limited studies on the annealing of TiO_2_ prepared by spatial ALD (sALD).

In this paper, TiO_2_ films are prepared by using sALD with TTIP and water precursors. The annealing temperature is varied, and its effect on the optical and crystalline properties of the films is investigated. Finally, the sALD TiO_2_ is applied to perovskite solar cells as a compact layer. The solar cell performance, such as the open circuit voltage (*V_oc_*), short circuit current density (J_sc_), fill factor (FF) and conversion efficiency (η), is also discussed.

## 2. Materials and Methods 

P-type 1–20 Ω-cm, CZ-type silicon (100) wafers and borosilicate glasses with a thickness of 0.2 mm were used as substrates. The glass substrates were ultrasonic-cleaned with propanol, alcohol and deionized water for 10 min each, and dried in nitrogen. The silicon wafers were cleaned using RCA standard procedures, followed by a dip in HF solution to remove natural oxide on the silicon surface, and finally they were dried with nitrogen. TiO_2_ thin films were prepared using a home-built sALD system (model Al_2_O_3_, Henghao, Taiwan) with TTIP (99.9999% purity, Aimou Yuan Scientific, Nanjing, China) and H_2_O as the titanium source and oxidant, respectively. The TTIP bubbler was placed in a gas cabinet equipped with a smoke detector. The vapor of the TTIP precursor was delivered through 1/4’ stainless steel tubing with VCR fittings to ensure safety. The reactor consisted of a substrate stage able to move back and forth and three precursor nozzles arranged in the order of H_2_O/TTIP/H_2_O. Each precursor nozzle was separated by a nitrogen curtain nozzle. The diameter of the nozzles was about 0.2 mm, and the gap between the injectors and substrate was 2 mm. The deposition area could be 15.6 cm × 15.6 cm. In this area, the error of the TiO_2_ film thickness was within 3%, evaluated from the nine-point measurement. The TTIP bubbler was heated to 70 °C to obtain a sufficiently high vapor pressure. Nitrogen (99.999% purity) was used as the carrier gas. The TTIP pipeline was heated to 75 °C to prevent vapor condensation. The temperature of the deionized water bubbler and delivery pipeline were kept to 25 and 30 °C, respectively. The substrate temperature was set to 110 °C. Nitrogen with flow rates of 400 and 2000 sccm were used as the carrier gas for TTIP and water, respectively, in order to take the precursor vapor out of the bubbler before it was diluted in 800 and 4000 sccm of nitrogen before injecting it into the substrate. A flow rate of 15,000 sccm was used for the nitrogen curtain. The detailed process parameters are listed in Table 1. After the film deposition, the samples were annealed in a furnace tube in nitrogen ambient at 350–750 °C for 30 min. The TiO_2_ films were deposited on the glass and silicon in order to investigate the effect of the annealing temperature on the optical and structural properties of the films. The thickness and refractive index of the films were determined using an ellipsometer (M-2000, J. A. Woollam Co., Inc., Lincoln, NE, USA). The crystalline structure of the films was characterized using a grazing incidence X-ray diffractometer (TTRAXIII, Rigaku Co., Tokyo, Japan) with an incident angle of 0.5° over the 2θ range of 20–60° using CuKα radiation (λ = 1.5405 Å), 45 kV cathode voltage and 40 mA cathode current. The chemical state and composition of the films were obtained by an X-ray photoelectron spectrometer (XPS, ESCALAB 250Xi, Thermo Fisher Scientific Co., San Jose, CA, USA) with an Al-Ka monochromatic source. The transmittance and reflectance of the samples were measured by a UV-visible spectrometer (MFS-630, Hong-Ming Technology, New Taipei, Taiwan). 

For the perovskite solar cell fabrication, 2 cm × 2 cm fluorine-doped tin oxide (FTO) glass substrates were cleaned with a detergent solution, deionized water, acetone and anhydrous ethanol for 10 min each. The substrates were further cleaned with UV ozone for 30 min, followed by the deposition of the sALD TiO_2_ layer with a thickness of ~8 nm. A ~50 nm, the SnO_2_ layer was then deposited on the sALD TiO_2_ by spin-coating a diluted SnO_2_ nanoparticle paste (2.7% in ultra-pure water) (Alfa Aesar, Shanghai, China) at 3000 rpm for 45 s, and dried at 150 °C for 30 min and sintered at 550 °C for 30 min in a muffle furnace (Michem instruments Co., Beijing, China). Formamidinium iodide (TCl America, Portland, OR, USA), methylammonium bromide (Aladdin, Shanghai, China), lead bromide (Sigma-Aldrich, St. Louis, MO, USA) and lead iodide (Alfa Aesar, Shanghai, China) were dissolved in a mixed solvent of *N*,*N*-dimethylformamide (DMF, Aladdin, Shanghai, China) and dimethyl sulfoxide (DMSO, Aladdin, Shanghai, China) (4:1 volume ratio) by a molar ratio of 1:1.15:0.2:0.2. Then, CsI (Sigma-Aldrich, St. Louis, MO, USA) previously dissolved as 1.5 mol stock solution in DMSO was added to the DMF/DMSO solution to achieve the Cs_0.1_(FA_0.83_MA_0.17_)_0.9_Pb(I_0.83_Br_0.17_)_3_ perovskite precursor solution, which was spin-coated on the SnO_2_ in a two-step process at 1000 rpm for 10 s and 6000 rpm for 25 s in a nitrogen glove box. Chlorobenzene (Aladdin, Shanghai, China) of 110 μL was dropped on the spinning substrate at 5 s before the second process finished. The substrates were then annealed at 100 °C for 1 h on a hotplate in order to obtain crystalline 3D perovskite films. After the substrates were cooled to room temperature, 50 μL of Spiro-MeOTAD (Lumtec, New Taipei, Taiwan) was spin-coated on the perovskite layers at 4000 rpm for 30 s. The devices were finalized by evaporating Au on the top of the Spiro-MeOTAD layer. The active area of perovskite solar cells was 0.5 cm × 0.5 cm. The cross-sectional images of the perovskite solar cells were obtained using a transmission electron microscope (TEM, CM200, Philips, Hillsboro, OR, USA). The photovoltaic performance was measured using a digital source meter (Model 2420, Keithley Instruments Inc., Cleveland, OH, USA) under an illumination of 100 mW/cm^2^ produced by a solar simulator at AM 1.5 G (Newport Oriel, Irvine, CA, USA). The light intensity was calibrated with a Si reference cell (Newport Corporation, Irvine, CA, USA) before the measurement. The J-V characteristics of PSCs were measured in forward (−0.1 to 1.2 V) and reverse (1.2 to −0.1 V) scan modes at a scan rate of 200 mV/s within a step of 20 mV.

## 3. Results

**Figure 1:**
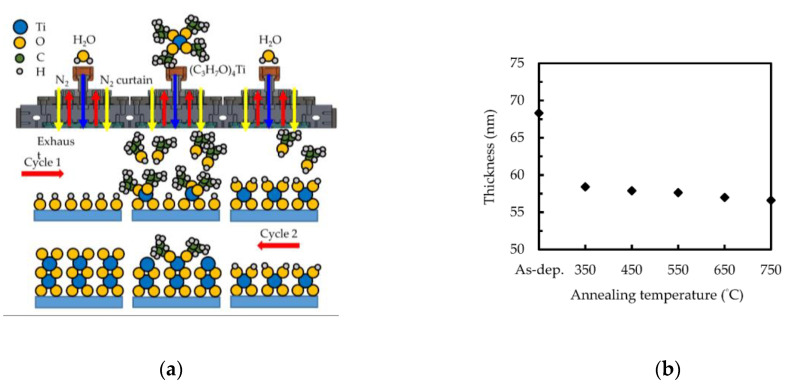
(**a**) Schematic diagram of the mechanism of sALD TiO_2_ deposition. (**b**) Thickness of the sALD TiO_2_ films before and after annealing at different temperatures.

Figure 1a shows a schematic diagram of the growth mechanism of sALD TiO_2_ films with TTIP/H_2_O precursors. On some substrates such as glass, the surface naturally carries hydroxyl groups. Otherwise, the surface will be hydroxylated when exposed to H_2_O for the first time. Excess precursor molecules are exhausted, and then the substrate moves to the TTIP precursor zone. Surface ligand exchange occurs between the hydroxyl group and TTIP, followed by the release of gaseous (C_3_H_7_)OH molecules. The chemical reaction can be described as [38]:

S–OH* + Ti(C_3_H_7_O)_4_ → S–O–Ti(C_3_H_7_O)_3_* + (C_3_H_7_)OH (1)

where S represents the substrate surface. Since TTIP only reacts with the surface hydroxyl groups but not with itself, a single layer of Ti(C_3_H_7_O)_3_ is formed. The substrate then moves to the H_2_O precursor area, resulting in the following reaction:

Si–O–Ti(C_3_H_7_O)* + H_2_O → S–O–Ti(OH)* + (C_3_H_7_)OH (2)

A monolayer of TiO_2_ is deposited after TTIP and H_2_O exposure. It is worth noting that one sALD cycle takes 3 s per cycle, while vacuum-based thermal or plasma ALD takes more than 1 min, including precursor injection, purge and exhaust. Figure 1b shows the thickness of the SALD TiO_2_ films (1000 cycles) as a function of the annealing temperature. The thickness of the as-deposited TiO_2_ film is 68.3 nm, which corresponds to a growth per cycle (GPC) of 0.68 Å/cycle. This value is similar to the values of vacuum ALD using O_2_-plamsa [39], ozone [40] or H_2_O oxidant [41], suggesting that the growth is mainly limited by the steric hindrance of TTIP instead of the growth sites created by the oxidizing agent. After annealing at 350 °C, the film thickness decreased to 60 nm, and a further increase in the annealing temperature caused a slight decrease in thickness. The obvious thickness drops at 350 °C may be due to the release of unreacted precursors in the film, which is consistent with the decomposition temperature of TTIP at 275 °C [42]. The slight decrease in thickness at higher temperatures is caused by the densification of the films.

**Figure 2:**
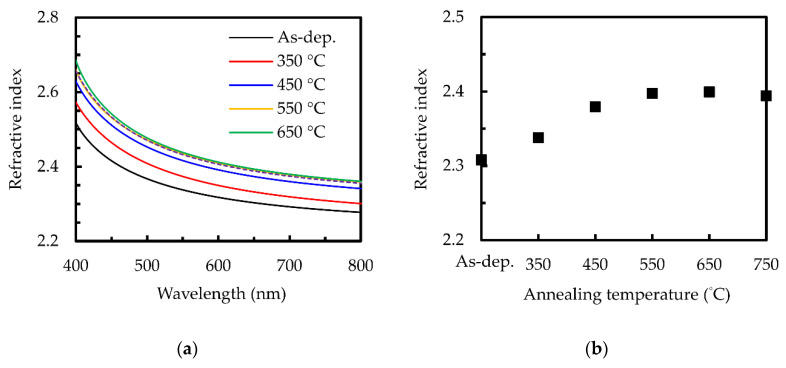
(**a**) Refractive index spectra and (**b**) refractive index at a 632 nm wavelength for the sALD films without and with various annealing temperatures.

Figure 2a shows the refractive index spectra of sALD TiO_2_ films at different annealing temperatures. For a comparison between different samples, we choose the refractive index with a 630 nm wavelength, as shown in Figure 2b, which is usually used for solar cell applications as it corresponds to the highest intensity of the solar spectrum. The refractive index of the film before annealing is 2.3 and seems to reach the maximal value of 2.4 as the annealing temperature reaches 550–750 °C. It is reported that the density of TiO_2_ films generally increases with the refractive index [43]. The films annealed at 550–750 °C have the highest refractive index, which is similar to those of high-quality PEALD TiO_2_ films with a corresponding film density of 3.8 g/cm^3^ [43]. In comparison, the sol-gel spin-coated TiO_2_ films have refractive indices typically under 2.2 [44,45], corresponding to a density of 2.95 g/cm^3^.

**Figure 3:**
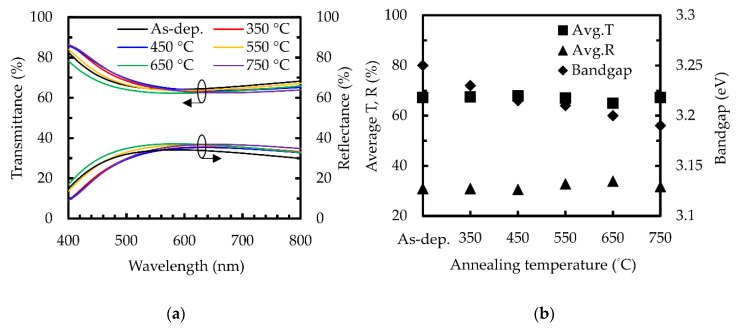
(**a**) Transmittance and reflectance of sALD TiO_2_ films with various annealing temperatures. (**b**) Average transmittance, average reflectance and band gap as a function of the annealing temperature.

Figure 3a shows the transmittance and reflectance spectra of the sALD TiO_2_ film at different annealing temperatures. The trend of the transmittance curves is opposite to that of the reflectance curves. This indicates that the change between the transmittance is mainly due to the reflectance, which is caused by the differences in the refractive index and thickness after annealing. Figure 3b shows the average transmittance, average reflectance and band gap for the sALD TiO_2_ films. The average transmittance and reflectance of the films range from 64.9–67.9% and from 30.5–33.8%, respectively. To further eliminate the disturbance caused by the sample thickness and reflection, we calculated the absorption coefficient of the films, which can be written as:

(3) $$\alpha \left(\lambda \right)=-\frac{1}{d}\ln\left[\frac{T\left(\lambda \right)}{1-R^{2}\left(\lambda \right)}\right]$$

where *α* is the absorption coefficient, *T* is the transmittance, *R* is the reflectance, *λ* is the wavelength and *d* is the film thickness. The magnitude of the absorption coefficient does not change significantly with the annealing temperature, and it stays at a low level of 10^3^ cm^−1^ at wavelengths greater than 500 nm for all the samples. However, the absorption coefficient of these sALD TiO_2_ films at shorter wavelengths (< 500 nm) is relatively higher, which is to say at the level of 10^4^ cm^−1^_,_ suggesting that the thickness of the films should still not be too thick for their use as a window layer of solar cells. The band gap of the films was obtained using the Tauc plot method [46]:

(*αhv*)*^n^* = A (*hv* − *E_g_*) (4)

where *E_g_* is the band gap, *hv* is the photon energy, A is a material constant and the exponent *n* characterizes the nature of the electron transition. For the indirect band gap *n* = 2, the value of the gap is obtained from the x-intercept of the extrapolated linear part of the graph (*αhv*)^1/2^ versus the photon energy, which is widely used in the literature for TiO_2_ [10,47]. The as-deposited film had the highest band gap, at 3.25 eV. When the annealing temperature increases from 350 to 750 °C, the band gap decreases from 3.23 to 3.18 eV. The band gap before annealing is consistent with that of amorphous TiO_2_. The decrease in the band gap with an increasing annealing temperature implies the structural change of the films. The samples annealed at below 750 °C have band gap values similar to anatase TiO_2_ (~3.2–3.25 eV), while the 750 °C-annealed sample shows a reduced band gap similar to rutile TiO_2_ [48].

**Figure 4:**
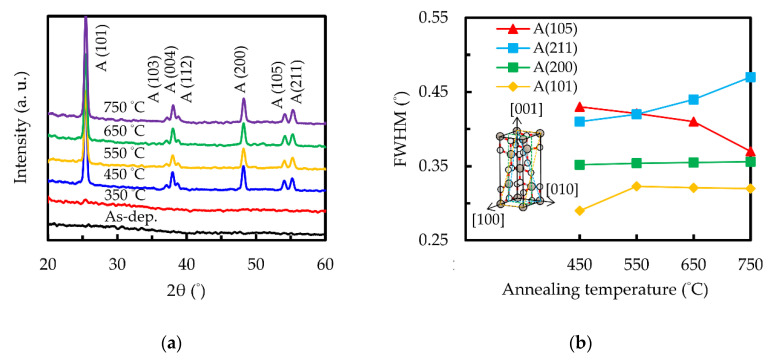
(**a**) X-ray diffraction patterns and (**b**) FWHM of the sALD TiO_2_ films without and with different annealing temperatures. The Inset indicates the crystalline planes of anatase TiO_2_.

To confirm the crystalline structure, the X-ray diffraction patterns of the sALD TiO_2_ films with different annealing temperatures are shown in Figure 4a. The peaks at 2θ = 25.3°, 36.9°, 37.8°, 38.6°, 48.1°, 53.9°and 55.1° correspond to (101), (103), (004), (112), (200), (105) and (211) anatase phases (JCPSD card #83-2243). The sample before annealing did not have any observable peaks. This is in good agreement with other studies using thermal ALD, showing that deposition below 200 °C results in amorphous TiO_2_ [49,50,51]. The film annealed at 350 °C seems to have a very weak peak at 25.3°, but it was difficult to distinguish from the background. The film structure changes from amorphous to polycrystalline anatase at 450 °C. When the annealing temperature increases to 750 °C, no other phases such as rutile (main peak at 2θ = 27°) are presented, indicating that the film maintains the polycrystalline anatase phase in a wide temperature range. The previously mentioned low band gap of the 750 °C-annealed sample is therefore not attributed to the formation of the rutile phase. Another possible reason for the low band gap is related to the sub-bandgap absorption caused by high temperature-generated defects, leading to localized states under the conduction band minimum and a shift of the absorption edge towards long wavelengths. Figure 4b shows the full width at half maximum (FWHM) of the diffraction peaks as a function of the annealing temperature. It can be seen that the FWHM of the (100) and (200) peaks do not change significantly. The FWHM of the (211) peak rises with an increasing annealing temperature, while the FWHM of the (105) peak decreases, implying that annealing temperature causes the (211) anatase phase to be gradually replaced by the (105) phase.

**Figure 5:**
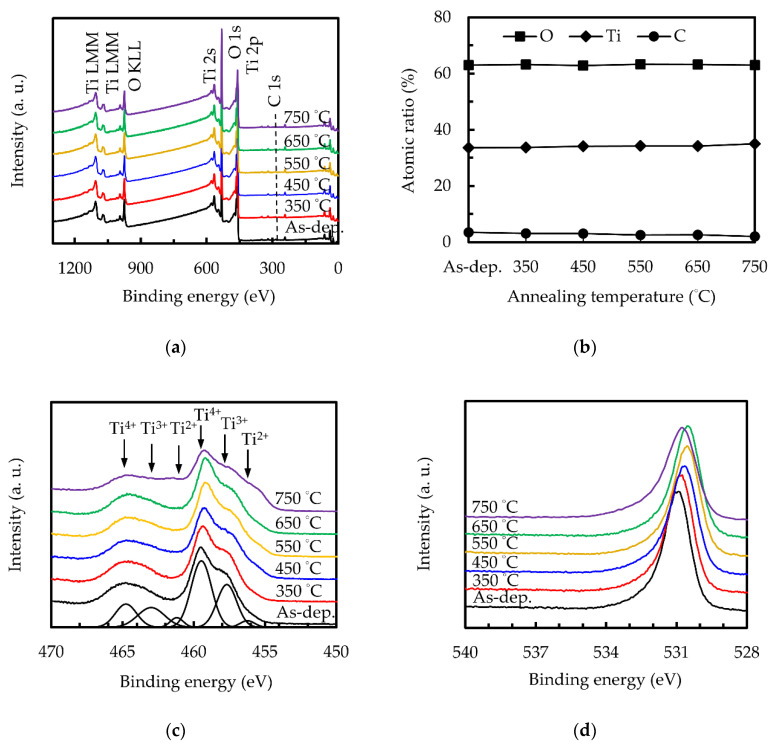
(**a**) XPS spectra, (**b**) atomic ratio, (**c**) Ti 2p, and (**d**) O 1s for the sALD TiO_2_ films.

Figure 5a shows the XPS spectra of sALD TiO_2_ thin films at different annealing temperatures. The Ti, O and C atomic ratios obtained from the XPS spectra are shown in Figure 5b, showing that all the films are oxygen-deficient with O/Ti ratios ranging from 1.87 to 1.79. The C atomic ratio decreases with an increasing annealing temperature. Figure 5c shows a high resolution of the Ti 2p peaks. All subsequent analyses are only based on the Ti 2p_3/2_ spin-orbit coupling state, but both the 1/2 and 3/2 states are fitted because the split is only about 5.7 eV, which leads to the overlap of Ti_3/2_^4+^ and Ti_1/2_^2+^. Compared with the 3/2 state, the area ratio of the 1/2 state is 0.43 ± 0.03. One can see that Ti 2p_3/2_ has three different oxidation states, which correspond to Ti^4+^ of 459 ± 0.2 eV, Ti^3+^ of 457.2 ± 0.2 eV and Ti^2+^ of 455.9 ± 0.2 eV, respectively [52,53]. It can be seen that the samples with an annealing temperature below 650 °C have similar results and that the proportion of Ti^4+^ increases slightly with an increasing annealing temperature, reaching the maximum at 550 and 650 °C. However, when the annealing temperature further increases to 750 °C, the composition of Ti^3+^ and especially Ti^2+^ increases significantly. In the O 1s spectrum, as shown in Figure 5d, the main peak shifts from 531.33 eV toward a lower energy. In the literature, the binding energy of 531.33 eV is usually related to hydroxyl groups, carbonate-like substances or other carbon-related impurities, or to oxygen-deficient titanium dioxide (e.g., TiO_*x*_, where 1.35 < *x* < 1.65) [54]. The first two explanations may be reasonable on the surface in the as-deposited state because it may have hydroxyl or carbon impurities that are adsorbed from the air or that remain in the structure as residues of incompletely reacted ALD precursors. The shift of the peak towards 530.2 eV corresponding to O^2−^ in TiO_2_ reveals the reduction of incompletely reacted ALD precursors and hence the densification after receiving the annealing treatment. The binding energy increases for the 750 °C-annealing can be explained by the increase in oxygen vacancies, arising from the desorption of oxygen.

**Figure 6:**
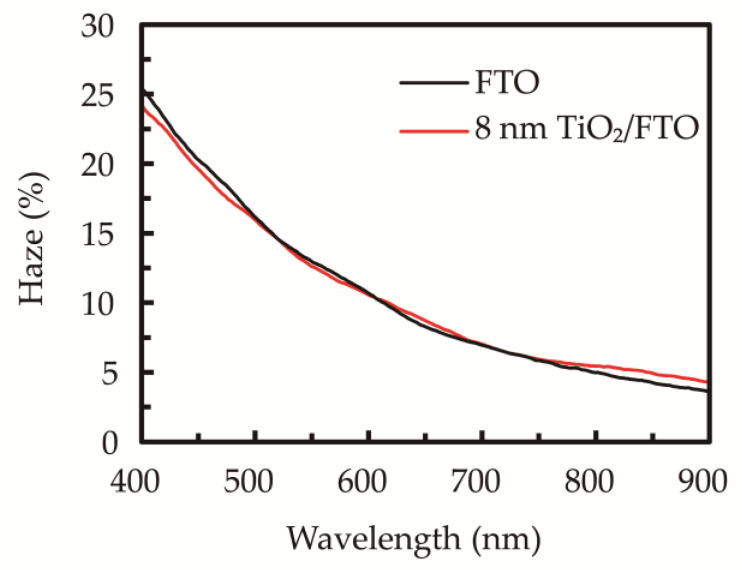
Haze spectra of the FTO substrates without and with the 8-nm sALD TiO_2_ layer.

From the above results, the 550 °C-annealed sALD TiO_2_ film having the highest compactness is considered for use as a compact layer of perovskite solar cells. The haze spectra of the FTO substrate without and with the 8-nm sALD TiO_2_ film is shown in Figure 6. The haze, defined as the percentage of light that is scattered at more than 2.5° from the incident light direction, is used as an indicator of the light diffusion. It is seen that the haze of the chemical-etched FTO is 25% at 400 nm, and it decreases to about 5% at 900 nm. The sALD TiO_2_ film on FTO does not significantly change the haze spectrum due to the high conformality and very thin thickness. The low degree of difference between the two spectra implies that the thin TiO_2_ deposition does not significantly change the optical property. 

**Figure 7:**
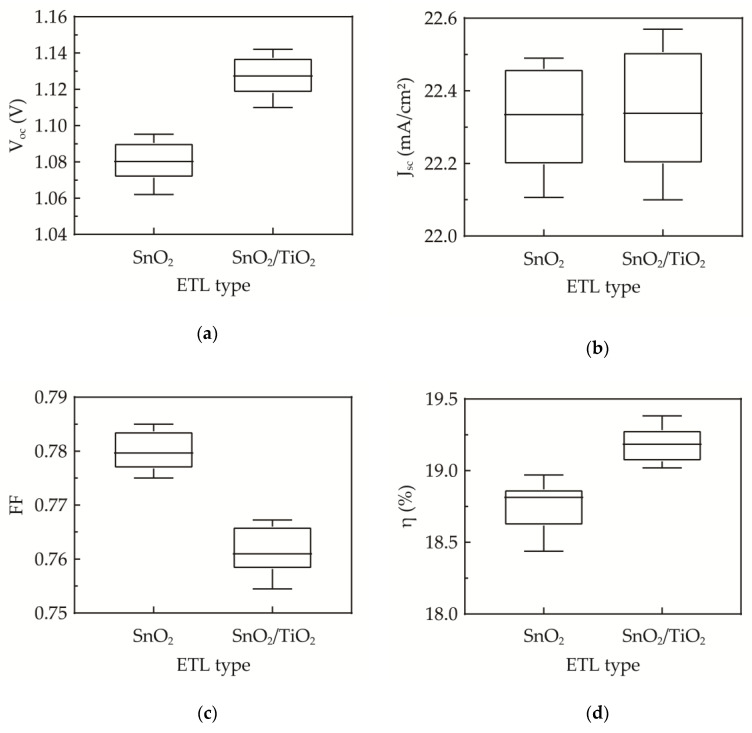
(**a**) *V_oc_*, (**b**) J_sc_, (**c**) FF and (**d**) η of the cells without and with the sALD TiO_2_ compact layer. The error bars show the range of data from ten devices fabricated under identical conditions.

Figure 7 shows the photovoltaic performance of the perovskite solar cells without and with the 8-nm sALD TiO_2_ compact layer measured in reverse scan. The error bars indicate the range of the data from ten cells fabricated under identical conditions. It can be seen that the cell with the sALD TiO_2_ compact layer shows a significant increase in *V_oc_* (Figure 7a). The lower *V_oc_* of the sol-gel SnO_2_ ETL alone implies that some of photo-generated holes are likely to pass through the ETL to recombine with electrons. This leads to an increase in the leakage current of the device. According to the following equation:

(5) $$V_{oc}=\frac{kT}{q}\ln\left(\frac{I_{sc}}{I_{0}}+1\right)$$

where k*T*/q is the thermal voltage, *I_sc_* is the short-circuit current and *I_0_* is the leakage current. Therefore, the hole-electron recombination at the FTO/ETL interface reduces the *V_oc_*. The sALD TiO_2_ used as a compact layer leads to enhanced hole blocking, a reduced leakage current and eventually an improved *V_oc_*, even at such a small thickness. The J_sc_ is not much influenced as shown in Figure 7b due to the similar refractive indices of TiO_2_ and SnO_2_, and the very thin TiO_2_ thickness. The amount of incident light is not much affected by the insertion of the sALD TiO_2_ layer. The slight decrease in FF as shown in Figure 7c could be explained by the increase in series resistance caused by the addition of the sALD TiO_2_ layer, and therefore it is important to keep the thickness of TiO_2_ as thin as possible. Overall, the conversion efficiency is significantly improved due to the *V_oc_* enhancement (Figure 7d). In the literature, most of the solution-based single-layer or double-layer ETLs give a *V_oc_* of 1.05–1.11 V [55,56,57,58], while the plasma-enhanced ALD or thermal ALD ETLs generally show a *V_oc_* of 1.09–1.11 V [59,60,61]. The sALD SnO_2_/TiO_2_ ETL in this study leads to an improved *V_oc_*. Furthermore, while the sALD and traditional vacuum-type ALDs have insignificantly different GPC values (~0.7 Å/cycle), it costs 3 s for one sALD cycle and at least 60 s in the case of the plasma or thermal ALD. For an 8-nm thickness of TiO_2_, sALD needs about 6 min to finish the deposition, whereas other ALDs require about 70 min. In this study, a high deposition rate sALD is used for preparing the very thin TiO_2_ compact layer. Although it is noted that the crystallinity of the 8-nm sALD TiO_2_ may not behave exactly as the 60-nm layer previously used for film characterization, the thin TiO_2_ compact layer leads to an improved *V_oc_* and conversion efficiency. The sALD is thus more advantageous in the application of perovskite solar cells.

**Figure 8:**
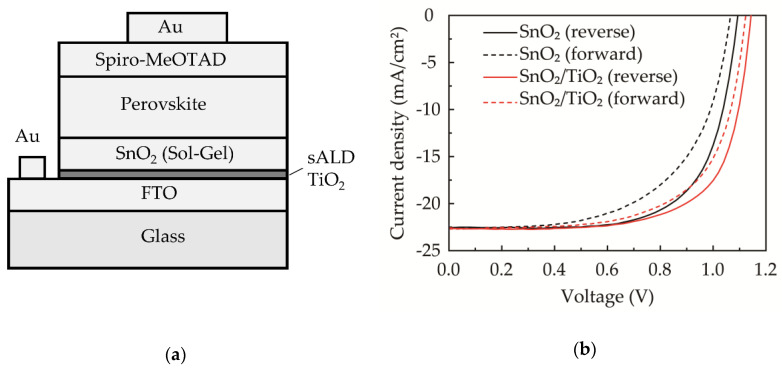
(**a**) Schematic of the structure of the perovskite solar cells. (**b**) J-V curves of perovskite solar cells without and with an 8-nm-thick sALD TiO_2_ compact layer.

**Figure 9:**
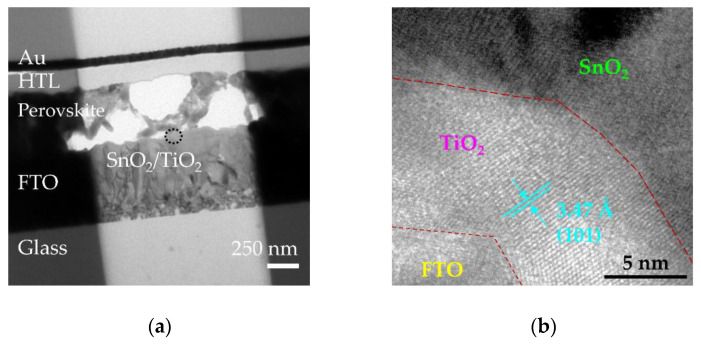
TEM images for (**a**) the perovskite solar cell and (**b**) the FTO/sALD TiO_2_/sol-gel SnO_2_ interface, with 3.47 Å d-spacing corresponding to the (101) plane of the tetragonal anatase TiO_2_ crystal structure.

Figure 8a shows the device structure, where the sALD TiO_2_ is added and inserted between the FTO and sol-gel SnO_2_ ETL in order to enhance hole blocking. The thicknesses of the FTO, sALD TiO_2_, sol-gel SnO_2_, perovskite layer, Spiro-MeOTAD and Au are about 600, 8, 50, 450, 150 and 65 nm, respectively. The corresponding cross-sectional TEM image for the device is shown in Figure 9a, and the high-resolution image at the interface region of FTO/sALD TiO_2_/sol-gel SnO_2_ is observed in Figure 9b, evidencing the very smooth interface and uniform (or conformal) TiO_2_ layer covering on FTO. The main lattice structure in the sALD TiO_2_ is evaluated to be 3.47 Å, which is indexed to the TiO_2_ (101) phase. Figure 8b compares the J-V curves for the highest efficiency perovskite solar cells without and with the sALD TiO_2_ compact layer. The corresponding external photovoltaic parameters are listed in Table 2. To quantify the magnitude of the hysteretic effect, the hysteresis index (*HI*), defined as *HI* = (*P_oc-sc_* − *P_sc-oc_*)/(*P_oc-sc_* + *P_sc-oc_*), is used, where $P_{oc-sc}=\int_{sc}^{oc}J_{R}\left(V\right)\Theta \left(J_{R}\right)dV$ and $P_{sc-oc}=\int_{oc}^{sc}J_{F}\left(V\right)\vartheta \left(J_{F}\right)dV$ are the values of the integral power output in the reverse and forward directions, respectively [62]. *Θ* is the Heaviside step function. It is found that the *HI* decreases by nearly 50% with the addition of the sALD TiO_2_ compact layer. Furthermore, the conversion efficiency of the perovskite solar cells was measured after fabrication and after one, three, five and seven days. The cells were stored in a nitrogen box. The cell efficiency measured at one day after fabrication was the highest, and the data are shown in Figure 8b. The cell efficiency degraded by about 5% when measured at seven days after fabrication. To further improve the stability, perovskite halides doped with Ni or Co might be a possible option, as suggested in ref. [63]. It is concluded that the sALD TiO_2_ with a high compactness, precise thickness control, high deposition rate and good step coverage demonstrates great potential for the applications of perovskite solar cells.

## 4. Conclusions

The TiO_2_ thin films are prepared using high growth rate sALD using TTIP and H_2_O precursors with different post-annealing temperatures. The 550–750 °C-annealed films have a refractive index of 2.4, close to the values of high-quality PEALD films and significantly higher than that obtained by the sol-gel spin-coating technique. All the TiO_2_ films have a low absorption coefficient at the 10^−3^ cm^−1^ level for wavelengths greater than 500 nm. The 550 and 650 °C-annealed TiO_2_ shows the highest Ti^4+^ proportion, while further increasing the annealing temperature to 750 °C leads to an increase in oxygen vacancies. Finally, the 550 °C-annealed sALD TiO_2_ compact layer with a very thin thickness of ~8 nm shows a significant improvement in *V_oc_*, demonstrating the great potential of sALD films for perovskite solar cell applications.

## Figures and Tables

**Table 1:** Deposition parameters of sALD TiO_2_ films.

Parameter	Value
Bubbler temperature (°C)	70
Substrate temperature (°C)	115
Substrate moving speed (cm/s)	15
Injector-to-substrate distance (mm)	2
H_2_O carry gas flow rate (sccm)	400
H_2_O dilute gas flow rate (sccm)	800
TTIP carry gas flow rate (sccm)	2000
TTIP dilute gas flow rate (sccm)	4000
Post-annealing temperature (°C)	350–750

**Table 2:** Performance of perovskite solar cells without and with the sALD TiO_2_ compact layer measured in forward and reverse scans.

Sample	Scan Mode	*V_oc_* (V)	J_sc_ (mA/cm^2^)	FF	η (%)
SnO_2_	Reverse	1.08	22.5	0.78	18.97
	Forward	1.05	22.51	0.70	16.51
SnO_2_/TiO_2_	Reverse	1.13	22.6	0.76	19.38
	Forward	1.11	22.51	0.71	17.74
